# Echocardiographic Assessment after Surgical Repair of Tetralogy of Fallot

**DOI:** 10.3389/fped.2015.00003

**Published:** 2015-02-02

**Authors:** Mario Carminati, Francesca R. Pluchinotta, Luciane Piazza, Angelo Micheletti, Diana Negura, Massimo Chessa, Gianfranco Butera, Carmelo Arcidiacono, Antonio Saracino, Claudio Bussadori

**Affiliations:** ^1^Department of Pediatric Cardiology and Adult with Congenital Heart Disease, IRCCS San Donato Hospital, Milan, Italy

**Keywords:** echocardiography, tetralogy of Fallot, right ventricular dysfunction, cardiac surgical procedures, strain rate

## Abstract

Surgical correction of tetralogy of Fallot is still one of the most frequently performed intervention in pediatric cardiac surgery, and in many cases, it is far from being a complete and definitive correction. It is rather an excellent palliation that solves the problem of cyanosis, but predisposes the patients to medical and surgical complications during follow-up. The decision-making process regarding the treatment of late sequel is among the most discussed topics in adult congenital cardiology. In post-operative Fallot patients, echocardiography is used as the first method of diagnostic imaging and currently allows both a qualitative observation of the anatomical alterations and a detailed quantification of right ventricular volumes and function, of the right ventricular outflow tract, and of the pulmonary valve and pulmonary arteries. The literature introduced many quantitative echocardiographic criteria useful for the understanding of the pathophysiological mechanisms involving the right ventricle and those have made much more objective any decision-making processes.

## Introduction

The complete surgical correction of tetralogy of Fallot (ToF) was first introduced in 1955 (1), and it is now used all over the world. In recent years, the surgical technique has gone through various improvements and due to the complexity and variability of the phenotypic presentation of the disease it is now performed with different approaches tailored to the patient’s anatomy, especially regarding the treatment of the right ventricular outflow tract obstruction and the related pulmonary valve stenosis. Initial ToF repair was mostly performed with transannular right ventricle (RV) outflow tract patch to relieve the obstruction. In most cases what we obtain after this surgical correction is far from a complete resolution of the disease. It is rather an excellent palliation that solves the problem of cyanosis, but predisposes the patients to subsequent interventions to treat the surgical sequelae. Nowadays, the most diffused surgical strategy is based on the presumption that the pulmonary annulus may be preserved and that a mixed lesion of moderate pulmonary stenosis and associated insufficiency is superior to the complete relief of obstruction and free pulmonary regurgitation.

In the long term, the residual pulmonary stenosis that remains after this conservative surgical approach and the free pulmonary regurgitation caused by the transannular patch graft used to enlarged the right ventricular outflow tract lead to the development of two pathophysiological conditions of the RV very different one from each other: RV hypertrophy and RV dilation.

Pulmonary valve regurgitation has been recognized as one of the most important risk factors for both right and left ventricular performance after the repair of ToF. Pulmonary regurgitation may be well tolerated for several years but, depending on its severity, it results in a progressive RV dilation and dysfunction. Long-standing chronic RV volume overload causes dilation of the tricuspid annulus that results in some degrees of tricuspid regurgitation. RV dilation and tricuspid regurgitation are important risk factors for the development of arrhythmias and possibly sudden death (2). Over time RV changes and remodeling secondary to volume and pressure overload reduce left ventricular function. This is most likely to be due to the alteration in the left ventricular and septal geometry secondary to RV dilation, post-surgical paradoxical systolic septal motion, and ventricular dyssynchrony (3). Retention of some pulmonary stenosis in the RV outflow tract as it is done with a surgical conservative approach may limit the jet width of pulmonary regurgitation and provides a protective RV ventricular hypertrophy that diminishes the deleterious effects of the retrograde pulmonary flow. Rao and colleagues reported their experience with 31 patients who underwent complete repair of ToF with preservation of the pulmonary valve. The data from this study demonstrate that pulmonary valve preservation is possible in most patients (28 over 31 enrolled) and the RVOT obstruction present right after surgery regresses as the valve participates in somatic growth (4). However, pulmonary valve-preserving repair in patients with severe hypoplastic pulmonary valves remains challenging and controversial (5, 6). Deorsola and colleagues proposed the preliminary results of an innovative procedure consisting in the implant of an injectable biological pulmonary valve, designed for right infundibular surgery in adults; in babies: the valves, shrunken to a smaller diameter, enable the implantation of a device wider than otherwise possible in young patients and once in the pulmonary position tends to expand to its original size following patient’s growth (7).

Imaging examination in adult post-operative patients with pulmonary regurgitation should be focused on the assessment of markers of RV function in order to identify the most appropriate timing for pulmonary valve replacement that remains controversial and is one of the most debated issues in the field (8). Several authors proposed cardiac magnetic resonance (CMRI) measurement of RV volumes as the most reliable indicators for pulmonary valve replacement: a RV end-diastolic volume >170 ml/m^2^ or a RV end-systolic volume >85 ml/m^2^ have been proposed (9) as a cut-off value for reoperation in order to obtain a substantial RV “normalization” after surgery. Other authors (10) considering the correlation between RV volumes, cardiac output, and exercise test changes after pulmonary valve replacement proposed a relatively more aggressive policy (RV end-diastolic volume <150 ml/m) (3) aiming to normalize the RV volumes, improve biventricular function, and submaximal exercise capacity after surgery (10).

## Echocardiographic Study of the Right Ventricle

The echocardiographic study of the RV after ToF surgery should include dimensional and volumetric measurements to determine the degree of segmental and global RV dilatation.

Echocardiographic quantitative parameter used to evaluate the right ventricular function are distinguished in geometrical and non-geometrical parameters: the first are based on bidimensional and three-dimensional measurements of RV volumes, the second rely on various technologies including M-mode, myocardial Doppler imaging, tissue Doppler imaging (TDI), and 2D strain.

### Geometrical parameters

**Figure 1:**
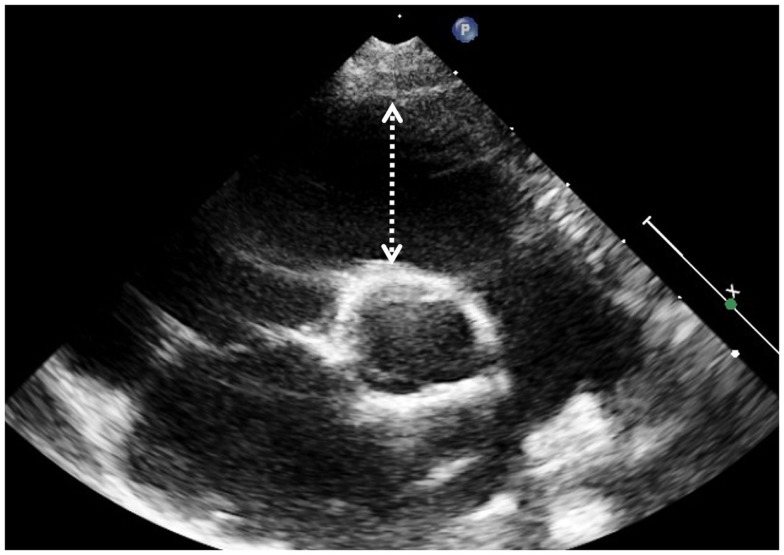
**The dotted arrow indicates the position where to measure the RVOT diameter**.

Indication for bidimensional measures of the RV have been proposed in various guidelines (11) and original articles (12). Dimensions and function of RV outflow tract in Fallot patients can be assessed by 2D echocardiography by measuring the fractional shortening of the RV outflow tract (FS-RVOT = [(end-diastolic length − end-systolic length)/end-diastolic length] × 100); measures are taken form the short axis at the level of the aortic valve along a line from the center of the ultrasound beam to the center of the aortic valve (Figure 1) (13). Another index commonly used in clinical practice is the fractional area change. This simplified index represents a “surrogate” measurement of RV ejection fraction (EF) and is expressed as a percentage change of diastolic and systolic area of the RV inflow measured on an apical 4-chamber view optimized for the RV. This method is based on geometric assumptions valid for the conical shape left ventricle that may not apply to the RV in general and especially to the RV in patients after ToF repair where the outflow tract is often markedly dilated. In a recent study, FS-RVOT was measured together with fractional area change in order to asses regional and global RV systolic function in adults after repaired ToF and compared to EF measured by CMRI: in the study the authors find that the optimal cut-off values to detect significant systolic RV dysfunction (defined ad RV EF <35% on CMRI) was a combination of fractional area change on 4-chamber view <30% and fractional shortening of the RV outflow tract <25% (sensitivity 79%, specificity 86%) (13).

The complex RV anatomy could be better investigated using three-dimensional echocardiography (3DE). This technology allows better assessment of the pulmonary valve’s morphology (14), and characterization of pulmonary flow (15) but underestimates RV volumes and EF (16, 17). Nonetheless, 3DE suffers by limitations such as low spatial and temporal resolution. A meta-analysis of 23 studies including a total of 807 patients (18) studied the accuracy of RV volumes and function determined by 3DE in comparison with CMRI and confirmed the tendency of 3DE to underestimate RV volumes and EF, especially when dealing with larger RV volumes (RV end-diastolic volume >200 ml) and younger patient (age <18 years) (19). Currently, 3DE RV assessment is probably not ready for routine clinical use in congenital heart disease patients with more than mild RV dilatation (19). Measurements of RV volumes are more reliable with CMRI that so far represents the gold standard for non-invasive quantitative assessment of the ventricular volumes (9). Cardiac MRI overcomes the echocardiographic limitation of poor image quality frequently encountered in patients who had several surgical procedures. However, the evaluation of the complex RV geometry requires a higher level of expertise and significant intra- and inter-operator variability for RV volume measurements on CMRI have been reported (20). Notwithstanding that, CMRI evaluation is recommended in all patients with repaired ToF and should be regularly used during clinical follow-up.

Cardiac MRI and echocardiography provide similar information useful for the optimal management of ToF patient. Echocardiography remains as the first-line diagnostic tool. Cardiac MRI is a valid alternative to echocardiography when ultrasound images do not have good resolution and when echocardiography measurements are borderline or ambiguous. Nowadays, echocardiography is superior in estimating gradients and pulmonary arterial pressure and offers optimal assessment of residual RV outflow tract obstruction and residual ventricular septal defect. Cardiac MRI is better than echocardiography for RV volumes and RV EF quantification, evaluation of the RV outflow tract obstruction and pulmonary artery conduits, and for pulmonary regurgitant flow estimation (21). Obstruction of the RV outflow tract observed in adult patients operated for ToF is more frequently caused by degeneration of the valved conduit surgically placed to correct pulmonary regurgitation. Optimal echocardiographic visualization of the site of obstruction is sometimes difficult to obtain because of the suboptimal windows due to previous sternotomy and conduit calcification. However, in patients who underwent percutaneous pulmonary valve implantation echocardiography almost always allows good visualization of the stent and of the prosthetic valve motion, and Doppler study of the blood flow through it. The most appropriate views to visualize the RV outflow tract and the pulmonary conduit are parasternal short axis and subcostal apical and short axis views. In addition, these views are ideal to study the integrity of the interventricular patch and to diagnose eventual residual interventricular shunts.

### Non-geometrical indexes

Echocardiographic indicators for studying velocity of displacement and deformation of the myocardium are aimed to a direct quantification of myocardial function not extrapolated from a change of shape and dimension.

**Figure 2:**
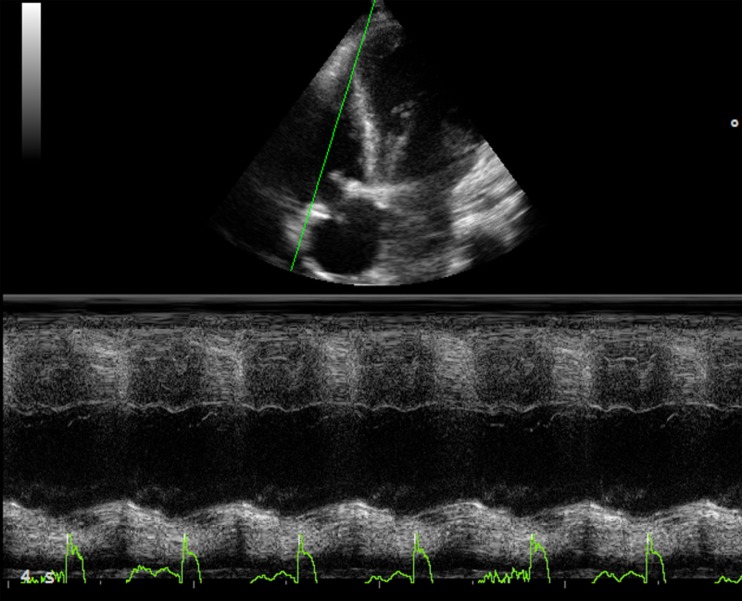
**Eighteen year-old man with a restrictive right ventricle, low value of TAPSE: 6 mm**.

**Figure 3:**
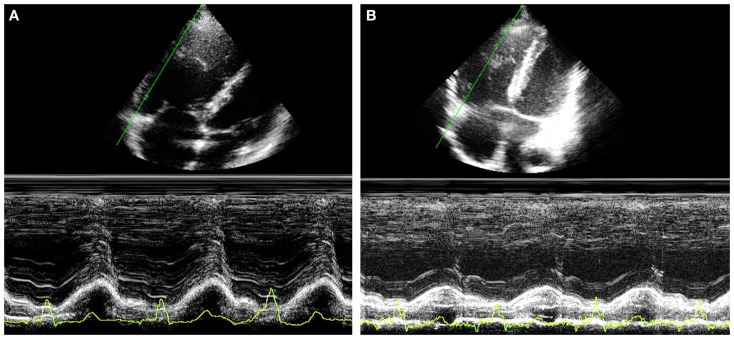
**Thirty year-old woman with severe pulmonary regurgitation**. **(A)** High value of TAPSE (22 mm) due to the volume overload. **(B)** Normalization of TAPSE 24 h after percutaneous implantation of a pulmonary prosthetic valve.

Tricuspid annular plane systolic excursion (TAPSE) measures the systolic excursion of the RV annular plane toward the apex. It is a very easy echocardiographic measurement, rapid to measure in a busy clinical setting, and is widely used in clinical practice even if its reliability is still controversial (22, 23). In adults values of TAPSE lower than 18 mm are suggestive of RV longitudinal dysfunction while in children the absolute value of TAPSE must be indexed to RV longitudinal diameter (TAPSE/RV longitudinal diameter ratio lower than 25% suggests longitudinal dysfunction) (24, 25). TAPSE has some important limitations. First of all, in patients with severe RV hypertrophy radial contraction becomes the prevalent direction of RV systolic deformation (26) and TAPSE, measuring the longitudinal deformation, may underestimate the real systolic function (Figure 2). Then TAPSE is strongly preload dependent and this may reduce its sensitivity regarding systolic function. In fact, in case of severe RV volume overload (large atrial septal defect, severe tricuspid, or pulmonary regurgitation) TAPSE may show high values masking mild systolic dysfunction (Figure 3). Several studies showed that there is a weak or no correlation between TAPSE and RV EF in ToF patients (27).

Tissue Doppler imaging technique measures the velocities of cardiac tissue and has been studied for the assessment of RV function. Tissue Doppler compared to blood flow Doppler reflects directly myocardial function and is less subject to preload changes. However, TDI is still Doppler based, and therefore its major limitation remains the angle dependency (28). Because of this the RV TDI analysis can be only applied at the tricuspid annular level or to the basal segments.

**Figure 4:**
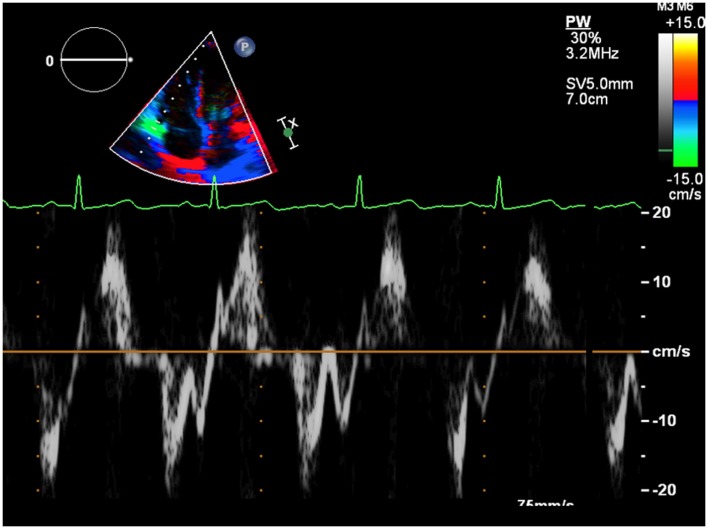
**The first rapid systolic wave represents the isovolumic contraction**. The pendency of the curve estimates the isovolumic acceleration.

Isovolumic acceleration time (IVA, Figure 4) is a TDI derived parameter that defines the myocardial acceleration during isovolumic contraction of tricuspid lateral annulus and has been proposed as a preload-independent indicator of RV contractility. It is calculated as the ratio of TDI derived peak myocardial velocity during isovolumetric contraction divided by the acceleration time. It has been validated in a variety of experimental and clinical settings. In a group of 124 patients after ToF repair IVA values measured at the tricuspid annulus and RV basal segments were lower in the affected population compared to normal controls and they correlated with the severity of pulmonary regurgitation (29). The two main limitations of using IVA derived values in the clinical setting are that IVA requires a good echocardiography equipment with high frame rate, and therefore has high variability among groups and centers, and that the physiological event to which IVA refers in the mechanics of the RV is not as well defined as it is in the left ventricle (30). Nevertheless, IVA (31), together with strain rate is the less load-dependent indexes available in echocardiography and it should be used for serial controls (32).

**Figure 5:**
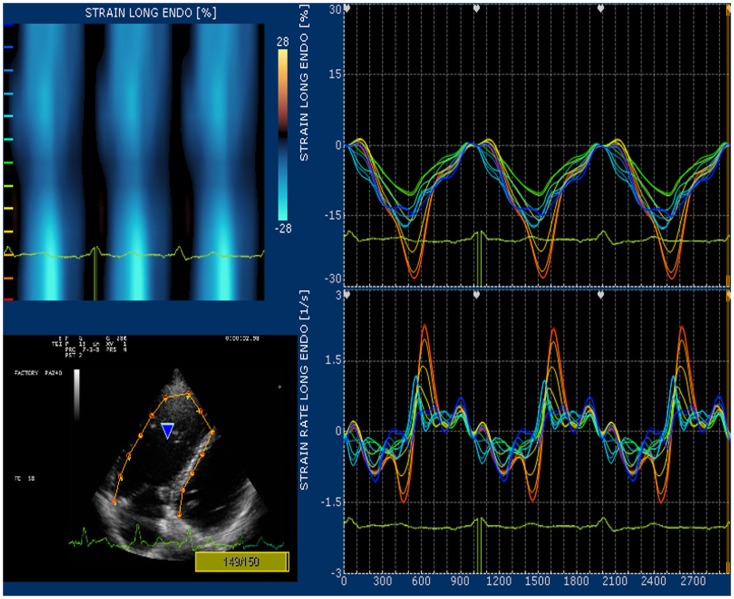
**High values of strain at the basal segment of lateral right ventricular wall**.

**Figure 6:**
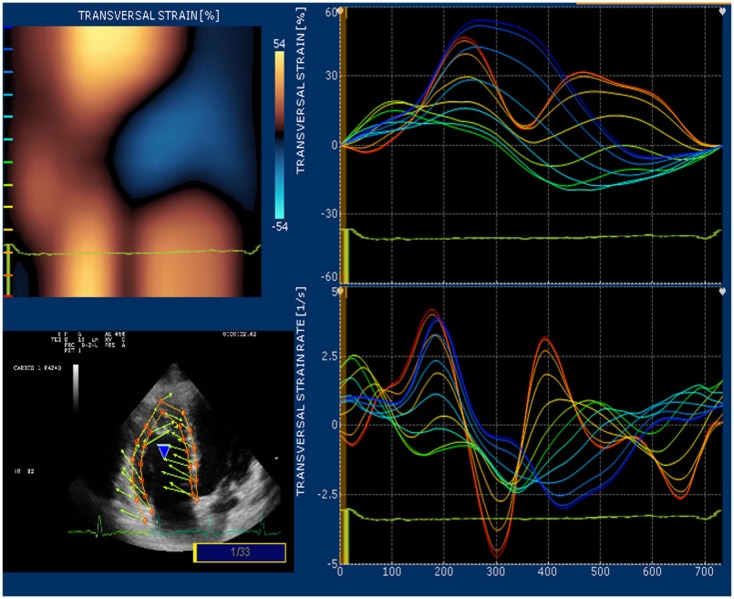
**High values of transversal strain in a ToF operated patient with restrictive RV**.

Strain and strain rate are largely used for direct quantification of systolic function. These newest 2D-based technologies overcome the limitation of Doppler angle dependence and are widely used to investigate left ventricular function. In many echocardiography laboratories, the experience on the strain and strain rate of the left ventricle is translated to evaluate RV function even if this use is considered “off label.” Since none of the available 2D strain software includes a template for the study of RV, in our lab we use the template designed for the left ventricular apical 4-chamber view and arbitrarily divide the RV lateral wall into basal, mid, and apical segments (32). Children and young adult operated for ToF present a various range of values for longitudinal strain and strain rate of right lateral wall and right interventricular septum. In case of severe pulmonary regurgitation with preserved RV systolic function, longitudinal strain is higher than normal at the basal lateral wall (Figure 5). On the other hand, patients with long-standing pulmonary regurgitation have decreased RV longitudinal strain that correlates with the degree of RV dilatation, severity of pulmonary regurgitation, and QRS duration (33). Depression of longitudinal strain is more evident in patient with dilated RV and stenosis of the pulmonary artery conduit. In this latter group of patients, a combination of pressure and volume overload causes stress on a previously dysfunctional RV with various degree of fibrosis and induces an evident afterload mismatch with very low values of longitudinal strain and strain rate. Low longitudinal strain is found also in severely hypertrophic restrictive RV, because the hypertrophy involves mainly the circumferential fibers and the systolic function of the RV is switched from a most prevalent longitudinal to radial deformation. In this case, a more correct quantification of RV systolic function can be done by measuring RV transversal strain (Figure 6). In patients after correction of ToF, global and RV free wall longitudinal systolic strain has been shown to continue to deteriorate despite unchanged RV EF, suggesting that regional wall motion assessment may detect early RV dysfunction (34). In patients with stenosis of the pulmonary conduit treated with percutaneous implant of a biological pulmonary valve, longitudinal strain of the RV increases significantly after pulmonary valve replacement but it never reaches normal values (35, 36).

## Doppler Study

Echocardiographic Doppler study in ToF as in many other cardiac diseases allows to obtain non-invasively hemodynamic information useful for decision making. Measure of pulmonary gradient with continuous wave Doppler allows to estimate the severity of pulmonic stenosis but could be sometimes not reliable in case of an anomalous anatomy (tunnel stenosis), a suboptimal alignment of the ultrasound beam with pulmonary flow, or in case of pulmonary hypertension and in pulmonary branch stenosis. Pulmonary hypertension and peripheral pulmonary stenosis lead to an underestimation of the severity of the stenosis (reduction of the anterograde peak gradient), and worsens pulmonary regurgitation. Underestimation of pulmonary stenosis should be suspected when the velocity of the tricuspid regurgitation exceeds consistently the velocity of the pulmonary flow. The suspect of pulmonary branch stenosis, which is more common than pulmonary hypertension in operated ToF patients, needs to be ruled out at the time of pulmonary valve replacement.

**Figure 7:**
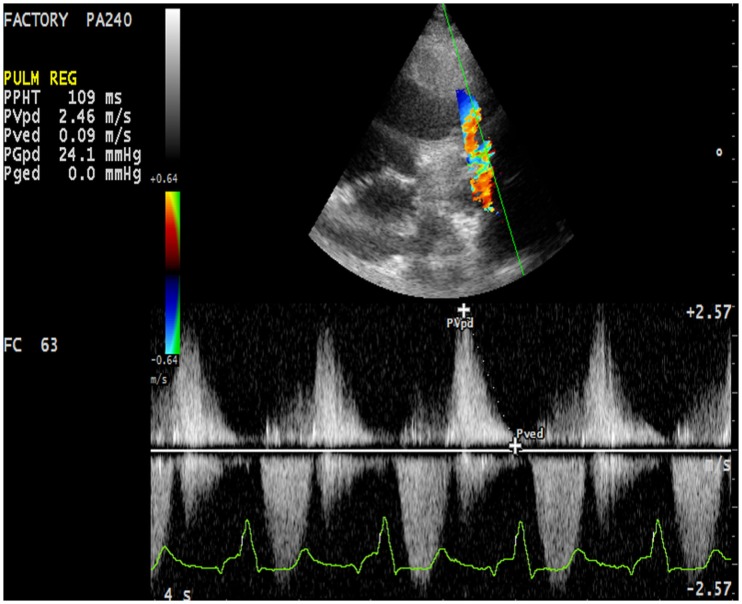
**Severe pulmonary insufficiency with early end and low values of pulmonary pressure half time (PHT)**.

First assessment of pulmonary regurgitation severity should be done with color Doppler: a severe pulmonary regurgitation is recognizable on color Doppler flow as a large retrograde flow that persists beyond the first half of diastole invading the RV outflow tract. After relieve of RV outflow tract obstruction with a transannular patch a severe pulmonary insufficiency appears as a “free floating” bidirectional flow across the pulmonary annulus. To record appropriately the color flow, it is recommended to set the color scale at the maximal pulse repetition frequency available in order to avoid turbulence overestimation. In severe pulmonary regurgitation, the most important determinants of the regurgitant volume are the compliance of the RV and the pulmonary artery, the size of the pulmonary branches, the pulmonary vascular resistance, and its changes throughout the cardiac cycle (37). Quantification of pulmonary regurgitation severity is assessed more precisely using spectral Doppler. The regurgitant velocity profile expresses the pressure gradient between the main pulmonary artery and the RV during diastole. If pulmonary diastolic pressures are normal, peak velocity is not higher than 1/ms. An indicator of severity is the precocity of the equalization of the two pressures; for example, in case of mild pulmonary regurgitation the regurgitant flow occupies the whole diastole while in patients operated for ToF the high regurgitant volume combined with a reduced compliant RV determinates an early interruption of the regurgitation (Figure 7). A quantitative assessment of pulmonary regurgitation severity is based on the deceleration velocity of the regurgitant flow known as pressure half time (PHT), the time in milliseconds taken to reach half of the pressure gradient. PHT could be easily measured using continuous wave Doppler. In a clinical validation study, PHT was demonstrated to be inversely correlated to the pulmonary regurgitant fraction measured by CMRI, and values of PHT under 100/ms were a highly specific and significant index of severe pulmonary regurgitation (38).

## Restrictive Right Ventricle

**Figure 8:**
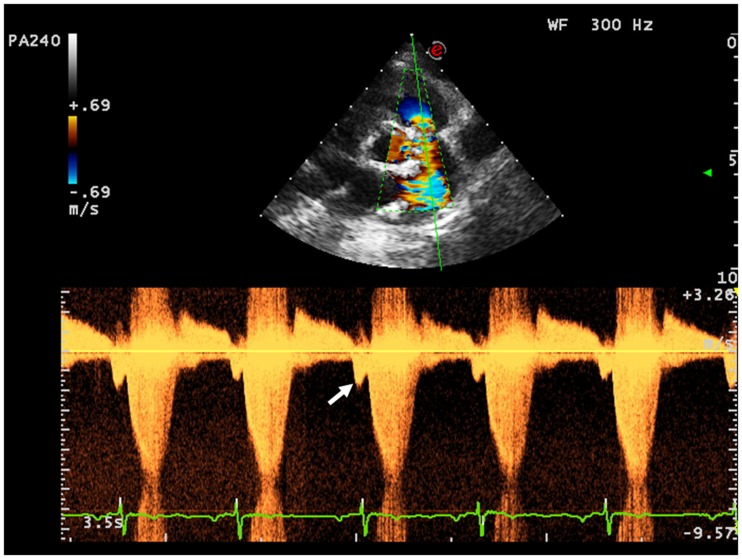
**The arrow indicates the end-diastolic forward flow**.

Right ventricle restrictive physiology is a condition observed in congenital heart diseases in which the RV systolic pressure is chronically elevated, and it is mostly encountered in adult patient operated of ToF. A restrictive physiology is typically seen in severely hypertrophied and fibrotic RV with normal sized or only mild ventricular dilatation due to the increasing myocardial stiffness. However, restrictive physiology could also be observed in severely dilated RV. In this case, the restrictive physiology should be intended as a manifestation of poor ventricular compliance that may occur at any stage of the RV remodeling after ToF surgery. Spectral Doppler may be used to identify this condition. Restrictive physiology may limit the degree of pulmonary regurgitation because of an increased mid-to-late diastolic pressure that overrides the pulmonary artery pressure. Therefore, the Doppler curve of the pulmonary regurgitation shows an early peak and an early end regurgitation. This gradient is defined the end-diastolic forward flow (EDFF) and appears just after the atrial contraction Figure 8. EDFF can be identified even in normal people during inspiration and for this reason, to define a RV restriction, the late diastolic anterograde flow should be recorded throughout the entire respiratory cycle: if respiration is not monitored during the exam the identification of EDFF in at least three consecutive cardiac cycles is considered pathognomonic of RV restriction. The limitation of the degree of pulmonary regurgitation in patients with restrictive RV has a protective effect on the RV by reducing the effect of volume overload (39). Severity of preoperative pulmonary stenosis and older age at time of intervention influence the post-operative RV hypertrophy and fibrosis and they represent the most important predisposing factors to a restrictive physiology of the RV (40). Patients with restrictive RV have a better performance at exercise test compared to other patients operated for ToF that have severe PR and do not have this diastolic dysfunction (41).

## Conclusion

Echocardiography is the exam most frequently performed in the follow-up of patients after complete ToF surgical correction. Use of validated echocardiographic indexes based either on conventional echocardiography and newest technologies such as 3DE and 2D strain allows us to integrate the usual subjective observational information with a new set of parameters useful to quantify RV function.

However, quantitative criteria represent an advantage when they are objectively repeatable, clinically reliable, and comparable with other quantitative values. This is not yet completely true for some of these echocardiographic indices applied to the RV, but in the near future it is reasonable to expect powerful clinical validation studies and improvements of the technologies in use.

Echocardiography with the development of new technologies such as 3DE, 2D strain, and myocardial Doppler imaging has made a great contribution to the follow-up of complex patients affected by congenital heart disease. Notwithstanding that, at present CMRI represents the gold standard for non-invasive quantitative assessment of the RV and should be regularly used in repaired ToF patients especially to evaluate those parameters in which CMRI is considered superior to echocardiography, such as RV volumes and RV EF.

The decisions about the complex diagnostic and therapeutic plans have to go through the analysis of all diagnostic techniques applied in these patients, as echocardiography, cardiopulmonary exercise tests, CMRI, CT, hemodynamic studies, electrophysiology, and biochemistry studies.

Furthermore, the introduction of more quantitative echocardiographic parameters for the evaluation of the RV has not decreased the number of cardiac catheterizations but it has indeed improved the added value of these invasive procedures probably making more accurate the indications to perform them.

The synergy between the information obtained by various methodologies in which the refinement of one technique also improves the level of information obtainable from the others is a further confirmation of the fact that the best diagnostic and therapeutic strategy can be identified only on the base of a multiparametric analysis that correlates data obtained from clinical and instrumental examinations.

## Conflict of Interest Statement

The authors declare that the research was conducted in the absence of any commercial or financial relationships that could be construed as a potential conflict of interest.
